# Inactivation of GPR30 reduces growth of triple-negative breast cancer cells: possible application in targeted therapy

**DOI:** 10.1007/s10549-012-1968-x

**Published:** 2012-01-31

**Authors:** Rainer Girgert, Günter Emons, Carsten Gründker

**Affiliations:** Department of Obstetrics and Gynecology, Georg-August-University Göttingen, Robert-Koch-Strasse 40, 37075 Göttingen, Germany

**Keywords:** Triple-negative breast cancer, Targeted therapy, GPR30, siRNA, Signal transduction

## Abstract

Triple-negative breast cancers lack estrogen receptor α (ERα), progesterone receptor, and do not overexpress human epidermal growth factor receptor 2 (Her-2). They are neither susceptible to endocrine therapy nor to a therapy using the anti-Her-2 antibody, trastuzumab. Therefore, an efficient targeted therapy is warranted. Triple-negative breast tumors frequently express membrane bound estrogen receptor G-protein coupled receptor (GPR30). As proof of principle, we analyzed the consequences of a knock-down of GPR30 expression on the growth regulation of triple-negative breast cancer cell lines. Cells of triple-negative breast cancer cell lines were transfected with siRNA against GPR30 or control siRNA, and cell growth was stimulated either with 10^−9^ M 17β-estradiol or 10^−6^ M 4-hydroxytamoxifen. Cell proliferation was measured using Alamar blue staining. Activation of c-Src and epidermal growth factor (EGF)-receptor was assessed using western blot. Expression of *c*-*fos* was quantified by reverse transcription polymerase chain reaction. Seven days after transfection with siRNA, GPR30 mRNA in triple-negative breast cancer cell lines MDA-MB-435 and HCC1806 was reduced by 74 and 90%, respectively. 10^−8^ M 17β-estradiol enhanced proliferation of MDA-MB-435 to 129.6 ± 5.4% of control (*p* < 0.05) and HCC1806 to 156.9 ± 15.4% of control (*p* < 0.05), respectively. 10^−6^ M 4-hydroxytamoxifen increased cell number of MDA-MB-435 to 121.0 ± 6.9% of control (*p* < 0.05) and HCC1806 to 124.5 ± 12.1% of control (n.s.), respectively. This increased proliferation by the two estrogenic compounds was completely prevented by knock-down of GPR30 expression in both cell lines. In control cells, activity of Src kinase was increased 3-fold by estradiol and 3.8-fold using 4-hydroxytamoxifen. Transactivation of the EGF-receptor was similarly increased in both cell lines by 17β-estradiol and 4-hydroxytamoxifen. Both compounds increased *c*-*fos* expression 1.5- and 3.1-fold, respectively. Knock-down of GPR30 expression completely abolished activation of all these signaling pathways responsible for enhanced proliferation. A pharmacological inhibition of GPR30 by specific small molecular inhibitors might prove to be an appropriate targeted therapy of triple-negative breast cancer in the future.

## Introduction

Breast cancer is the most frequent malignancy in women. Endocrine therapy with the anti-estrogen tamoxifen or aromatase inhibitors achieves an overall survival of about 82% of patients after 8 years of treatment [1]. A subgroup of tumors expressing neither ERα nor progesterone receptor and not overexpressing Her-2 accounting for 15–20% of all breast tumors is called triple-negative breast cancer. These tumors, not susceptible to endocrine therapy, are currently treated with conventional chemotherapy. The death rate of patients with triple-negative breast cancer is double as high as in the case of ERα-positive tumors [2].

Triple-negative breast cancers frequently carry mutations of the BRCA1 gene, for this reason, they turn out to be sensitive to platinum compounds. The combination of platinum and the epidermal growth factor (EGF)-R antibody Cetuximab increased the response rate from 30 to 49% [3]. Inhibitors of poly-ADP-ribose polymerase (PARP) were also found to be promising in triple-negative breast cancer [4, 5].

In MDA-MB-435 and MDA-MB-231 cells, lacking detectable expression of ERα, Tsai et al. [6] observed a rapid phosphorylation of protein kinase Akt at Ser^473^ after stimulation with 17β-estradiol. Adenylate cyclase activity was increased in MCF-7 breast cancer cells by 17β-estradiol within minutes leading to an activation of protein kinase A [7]. An activation of the MAP-kinase extracellular signal-regulated kinase (Erk) was also observed after short time stimulation of MCF-7 breast cancer cells with 17β-estradiol [8, 9]. It was assumed that an estrogen receptor resides at the plasma membrane [10]. Finally, the G-protein coupled receptor, GPR30, was identified to be responsible for most of the non-genomic signaling events of 17β-estradiol [11, 12]. Binding of 17β-estradiol to GPR30 leads to a dissociation of the heterotrimeric G-protein complex. The βγ-subunit activates the tyrosine kinase Src [13]. Subsequently, EGF from the extracellular matrix elicits the autophosphorylation of the EGF-receptor leading to the activation of the ras-MAP-kinase pathway [14].

GPR30 has been proposed to be an excellent new therapeutic target for the treatment of triple-negative breast cancer [15].

In addition to 17β-estradiol, selective estrogen receptor modulator, tamoxifen, and complete ERα antagonist, fulvestrant, bind to GPR30 and induce adverse effects in breast cancer cells [11].

The experiments described in this report were performed to elucidate the role of GPR30 in the proliferative response of triple-negative breast cancer cells to 17β-estradiol and anti-estrogen 4-hydroxytamoxifen. For this purpose, GPR30 expression was reduced in two triple-negative breast cancer cell lines using specific siRNA. The consequences of the knock-down of GPR30 expression were analyzed at several points along the signaling pathway of GPR30 after stimulation with either 17β-estradiol or 4-hydroxytamoxifen. The enhancement of proliferation of the triple-negative cell lines by 17β-estradiol or 4-hydroxytamoxifen was completely prevented by the knock-down of GPR30.

## Materials and methods

### Reagents

17β-Estradiol (E2), 4-hydroxytamoxifen, insulin, and transferrin were purchased from Sigma-Aldrich (Deisendorf, Germany). siRNA for GPR30 and non-specific control siRNA were obtained from SantaCruz Biotech (Santa Cruz, CA).

### Cell lines

MDA-MB-435 [16] was purchased from ATCC (Manassas, VA) and maintained in phenol red-free DMEM (Biochrom, Berlin, Germany) supplemented with 2 mM glutamine, 6 ng/ml insulin, 10 ng/ml transferrin, penicillin (50 U/ml), streptomycin (50 μg/ml) from Gibco (Paisley, UK), and 5% fetal bovine serum (Biochrom, Berlin).

Breast cancer cell line HCC1806 was purchased from ATCC (Manassas, VA). Cells were maintained in phenol red-free MEM with Earle’s salts supplemented with 5% fetal calf serum (FCS, Biochrom, Berlin), 2 mM glutamine, and 50 U/ml penicillin/streptomycin (Gibco, Paisley, UK).

### Transfection with siRNA

4 × 10^5^ Cells of each triple-negative breast cancer cell line were seeded in 2 ml growth medium into 25 cm^2^ culture flasks and grown to 80% confluence.

GPR30 siRNA (sc-60743) and nonspecific control siRNA (sc-37007) from SantaCruz (Santa Cruz, CA) were dissolved in RNAse-free water at a concentration of 10 μM. Sixteen microliters of each siRNA were diluted in 400 μl transfection medium (sc-36868). In addition, 32 μl transfection reagent (sc-29528) was added to another 400 μl transfection medium. Equal volumes of the diluted siRNA and transfection reagent were mixed and incubated at room temperature for 45 min.

Growth medium was removed from the culture flasks, and 2 ml transfection medium (sc-36868) was added to the cells. After 10 min at 37°C, transfection medium was replaced by the mixture of siRNA and transfection reagent, and the transfection was started. After 6 h, 2 ml of a growth medium containing a twofold concentration of FCS and antibiotics was added to the transfected cells. After further 18 h, the remaining siRNA was aspirated and cells were grown in normal culture medium until transfected cells were used for analysis.

### RT-PCRs

RNA of the transfected breast cancer cells was purified using the RNeasy-kit (Qiagen, Hilden, Germany).

200 ng of each RNA was transcribed using 400 U Superscript reverse transcriptase (Invitrogen, Karlsruhe, Germany) in the presence of 0.5 μM oligo-dT primer for 60 min at 37°C. Five microliter of the resulting cDNA was amplified with 1 U Taq polymerase (Peqlab, Erlangen, Germany) in the presence of 200 μM dNTPs and 200 nM of the appropriate primers.

- GPR30: primer A: AGTCGGATGTGAGGTTCAG
- Primer B: TCTGTGTGAGGAGTGCAAG
- *c-fos*: primer A: GAGATGGAGATCGGTATGGT
- Primer B: CAGGTCTGAATCAGTGCCTT

Optimal PCR conditions for each gene were ascertained, guaranteeing that generation of the PCR products was in the exponential phase. Therefore, cDNA of GPR30 was amplified by 28 cycles and *c-fos* by 32 cycles. As reference, the RNA of the ribosomal protein L7 was amplified by 20 cycles.

PCR products were separated in a 2% agarose gel (Type IV, special high EEO, Sigma Chemicals, Steinheim, Germany) in 0.5× TBE buffer at 100 V for 30 min. Gels were stained in ethidium bromide (2 μg/ml) for 30 min and photographed on a transilluminator using a CDS camera (TD20, Kodak, Rochester).

### Proliferation assays

The proliferation assays for 17β-estradiol and 4-hydroxytamoxifen were performed in phenol red-free medium supplemented with charcoal depleted serum (CD-FCS) as previously described [16]. CD-FCS was prepared according to the procedure described by Stanley et al. [17].

In brief, 2,000 cells/well were seeded in 100 μl CD-FCS medium (10% CD-FCS) into 96-well plates. After attachment of the cells, 100 μl CD-FCS medium containing either vehicle (control) or 17β-estradiol (10^−9^ and 10^−8^ M) or 4-hydroxytamoxifen (10^−7^ and 10^−6^ M) were added to four replicate wells for each concentration.

Cells were grown for 7 days at 37°C, 5% CO_2_, and saturated humidity. Cell number was determined by a colorimetric method using Alamar blue (Biosource, Solingen, Germany) [16].

Proliferation assays were performed at least three times in quadruplicates with different passages. Means and standard deviations of the optical density (OD) of the replicates were calculated.

### Assays of GPR30 signaling

For analysis of GPR30 signal transduction pathway, 10^6^ cells/well were plated in serum-free culture medium into 6-well plates (35 mm). Cells were serum starved for 24 h to synchronize the 17β-estradiol-starved cells in G_0_-phase. Serum starved cells were stimulated with 10^−8^ M 17β-estradiol or 10^−6^ M 4-hydroxytamoxifen for 10 or 20 min. Cells were harvested and cell pellets lysed in 100 μl Cell lytic M (Sigma, Deisendorf, Germany), supplemented with protease inhibitor (Sigma, Deisendorf, Germany) and phosphatase inhibitor (Sigma, Deisendorf, Germany).

### Western blots

Lysates of cells were cleared at 15,000g for 5 min, and the protein concentration in the supernatant was determined using the method of Bradford. 50 μg of each sample was separated in a 7.5% polyacrylamide gel, blotted on PVDF-membrane, and sequentially detected with rabbit-anti-human primary antibodies: anti-phospho-Src (2113), anti-Src (2109), anti-phospho Tyr^1173^EGF-receptor (324864) from Calbiochem (Darmstadt, Germany), anti-EGF-receptor antibody (2235) from Epitomics (Hamburg, Germany) and anti-actin from Sigma Chemicals (Deisendorf, Germany). After washing in TBST, blots were incubated with a 1:20.000 dilution of horseradish peroxidase-conjugated goat-anti-rabbit antibody (ECL, GE-Healthcare, Freiburg, Germany). After washing, blots were incubated with a chemoluminescence reagent and exposed to X-ray film (BiomaxMR, Kodak, Rochester, USA). Densitometric evaluations of the protein bands were normalized to actin.

### Densitometric evaluation of PCR products

The bands of the PCR products were photographed using a CDS camera and evaluated by the Digital science 1D-software (Kodak, Rochester, USA). Values of the reverse transcription polymerase chain reaction (RT-PCR) products were normalized to the ribosomal protein L7.

### Statistical analysis

The data were tested for significant differences by one-way analysis of variance followed by Student–Newman–Keuls’ test for comparison of individual groups, after a Bartlett test had shown that variances were homogenous.

## Results

### Degradation of GPR30 mRNA by the treatment with siRNA

**Fig. 1:**
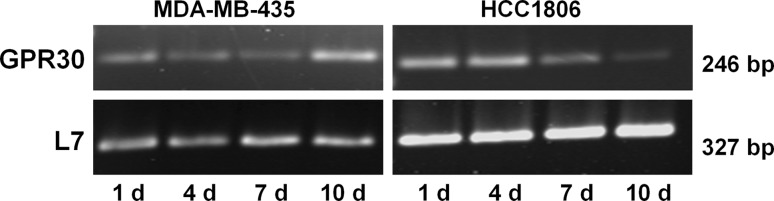
Knock-down of GPR30 expression in triple-negative breast cancer cell lines. MDA-MB-435 and HCC1806 were transfected with siRNA specific for GPR30. mRNA of the cells was extracted 1, 4, 7, and 10 days after transfection, transcribed into cDNA and amplified by PCR using specific primers. L7, a ribosomal housekeeping gene, was amplified to prove the presence of equal amounts of RNA in each PCR reaction of the respective cell line. Representative results from three independent transfections

Triple-negative breast cancer cell lines MDA-MB-435 and HCC1806 were transfected with either non-specific siRNA (control; −si) or with siRNA specific for GPR30 (+si). Expression of mRNA for GPR30 was analyzed using RT-PCR at various time points after transfection in order to track the time course of the decline of GPR30 mRNA expression in both cell lines. Treatment with non-specific siRNA did not change expression of GPR30 mRNA (data not shown). In MDA-MB-435 cells transfected with GPR30 siRNA, the amount of GPR30 mRNA was gradually decreasing with growing time of exposure to siRNA. m-RNA for GPR30 was lowest 7 days after transfection with siRNA and started to increase again on day 10 of siRNA treatment (Fig. 1). Seven days after transfection of MDA-MB-435 cells, expression of GPR30 mRNA was reduced to 26%.

In HCC1806 cells, expression of GPR30 mRNA decreased more slowly after transfection. Ten days after transfection, the amount of GPR30 mRNA was minimal and accounted for 10% of the expression in non-treated HCC1806 cells. L7 expression was unaffected by the treatment with GPR30 siRNA.

### Inactivation of GPR30 slows down proliferation of triple-negative breast cancer cells

**Fig. 2:**
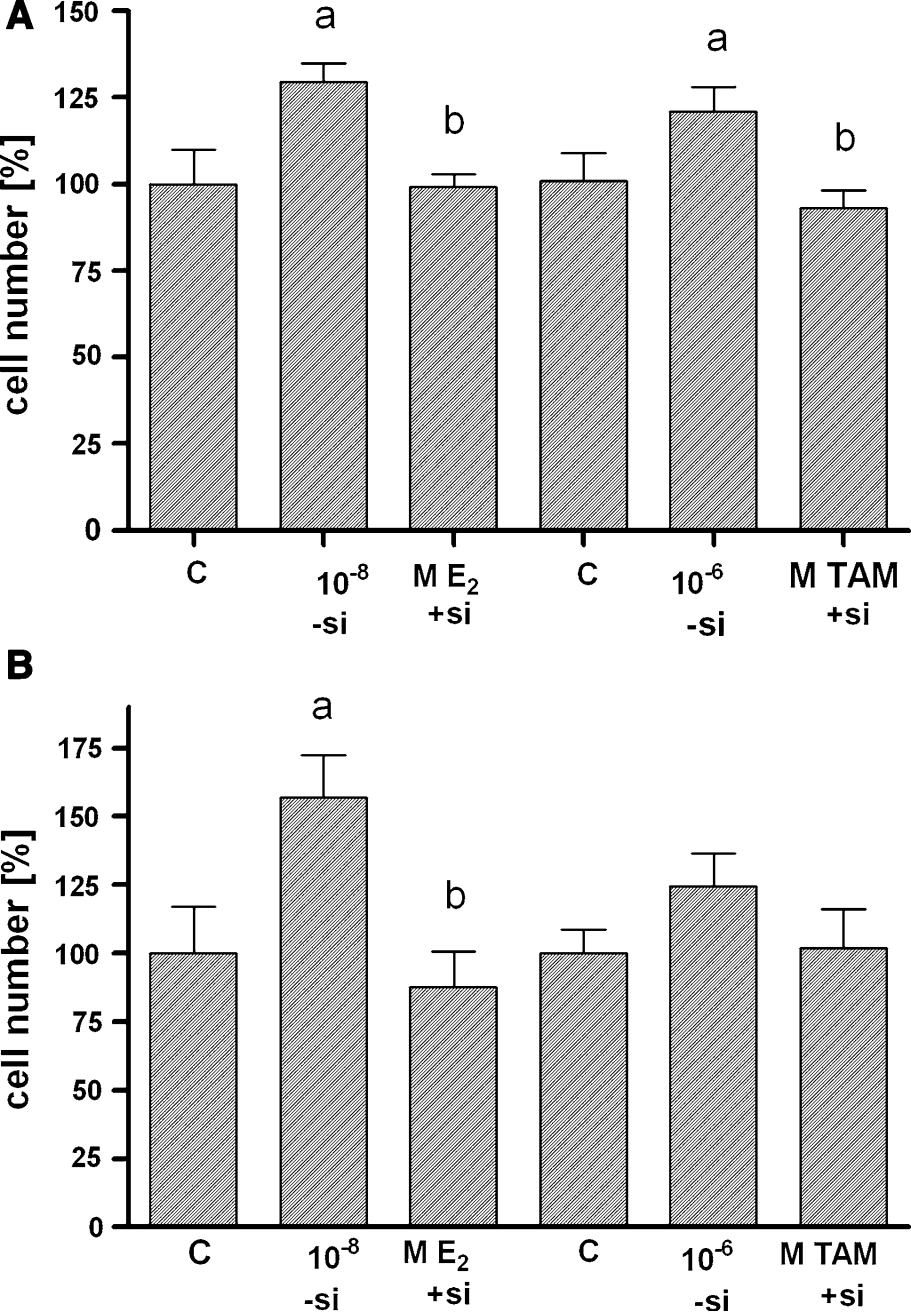
Inhibition of proliferation of triple-negative breast cancer cells after knock-down of GPR30 expression. **a** MDA-MB-435, **b** HCC1806. Cells transfected with non-specific siRNA (−si) or with GPR30siRNA (+si) were grown for 7 days in culture medium supplemented with 10% charcoal-treated FCS either in the absence or presence of 10^−8^ M 17β-estradiol or 10^−6^ M 4-hydroxytamoxifen. Cell number was evaluated in microwell plates by a colorimetric assay using Alamar blue. ODs measured in the non-stimulated wells (control) were set 100%. The ODs estimated in the stimulated wells were divided by the values of the control well to give the relative cell number in % achieved under the indicated conditions. Doubling times of cell lines in hormone-depleted medium (control cells) were: MDA-MB-435: ~48 h; HCC1806: ~36 h. Data are mean values and SE of three independent experiments with four replicates. **a** *p* < 0.05 versus control, **b** *p* < 0.05 versus −si

Proliferation of triple-negative breast cancer cell lines MDA-MB-435 and HCC1806 could be stimulated by 17β-estradiol and 4-hydroxytamoxifen (Fig. 2a, b). To test whether GPR30 is involved in the growth promoting effect of these two compounds, proliferation tests were performed with cells pretreated with siRNA against GPR30 (+si). After treatment of MDA-MB-435 control cells (−si) with 10^−8^ M 17β-estradiol, the cell number increased to 129.6 ± 5.4% of control (C = 100%; *p* < 0.05). The 17β-estradiol-induced increase was completely obviated by knock-down of GPR30 (+si; *p* < 0.05). After treatment of MDA-MB-435 control cells (−si) with 10^−6^ M 4-hydroxytamoxifen, the cell number increased to 121.0 ± 6.9% of control (C = 100%; *p* < 0.05). Knock-down of GPR30 expression completely suppressed the effect of 4-hydroxytamoxifen on proliferation of MDA-MB-435 cells (+si; 93%; *p* < 0.05) (Fig. 2a). In the HCC1806 breast cancer cell line, treatment with 10^−8^ M 17β-estradiol increased the number of control cells (−si) to 156.9 ± 15.4% of control (C = 100%; *p* < 0.05). This growth stimulation was completely prevented by knock-down of GPR30 expression (+si; 82%; *p* < 0.05). After treatment of HCC1806 control cells (−si) with 10^−6^ M 4-hydroxytamoxifen, the cell number increased to 124.5 ± 12.1% of control (C = 100%; n.s.). Knock-down of GPR30 expression completely suppressed the growth stimulation by 4-hydroxytamoxifen (+si; 97%; n.s.) (Fig. 2b).

### Impact of GPR30 knock-down on signal transduction of GPR30

**Fig. 3:**
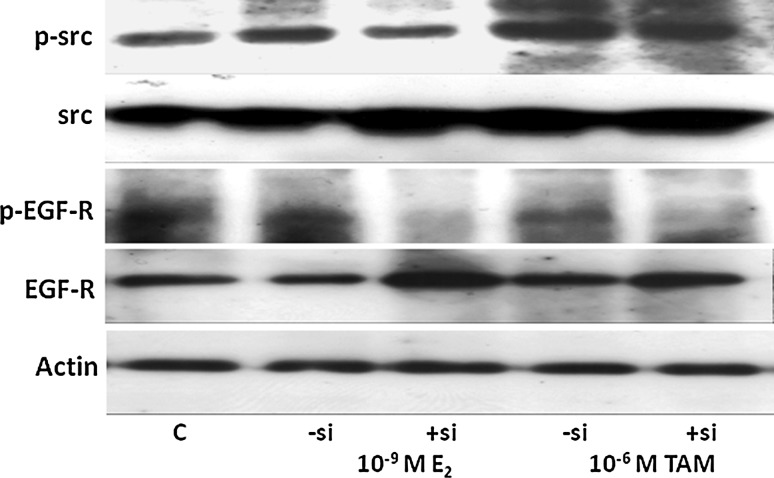
Disruption of signal transduction of GPR30 in the siRNA-treated cells of the triple-negative breast cancer cell lines MDA-MB-435. Cells transfected with siRNA (+si) and control cells (−si) were stimulated for 10 min with either 10^−9^ M 17β-estradiol or 10^−6^ M 4-hydroxytamoxifen. Cells were lysed, proteins separated in a polyacrylamide gel, blotted onto a PVDF-membrane, and the indicated proteins were sequentially detected with antibodies against phospho-src (panel 1), total-src (panel 2), phospho-EGF-receptor (panel 3), total EGF-receptor (panel 4), and actin, as housekeeping gene (panel 7). Representative results of three independent preparations

Stimulation of GPR30 by 17β-estradiol is known to increase activity of the non-receptor tyrosine kinase Src. Treatment of MDA-MB-435 cells with 10^−9^ M 17β-estradiol for 10 min clearly increased phosphorylation of Src at Tyr^416^ about threefold (Fig. 3, upper panel, lane 2). In MDA-MB-435 cells transfected for 7 days with GPR30 siRNA, the increase of Src phosphorylation by 17β-estradiol was completely abolished (lane 3). After treatment with 10^−6^ M 4-hydroxytamoxifen for 10 min phosphorylation of Src at Tyr^416^ increased by a factor of 3.8 ± 0.6. As seen for 17β-estradiol, inactivation of GPR30 by means of siRNA clearly obviated the activation of the kinase Src by 4-hydroxytamoxifen (Fig. 3, upper panel, lane 5).

Next step analyzed in the signal transduction of GPR30 was the transactivation of the EGF-R. In the course of GPR30 signaling, certain membrane bound matrix metalloproteases are activated and release EGF from the extracellular matrix. The degree of transactivation of the EGF-receptor was determined by detecting the amount of EGF-receptor phosphorylated at Tyr^1173^ on western blots. Phosphorylation of the EGF-receptor slightly increased after 10 min stimulation of MDA-MB-435 cells with 10^−9^ M 17β-estradiol (Fig. 3, panel 3, lane 2). In cells transfected with siRNA against GPR30 (+si), the phosphorylation status of the EGF-receptor was much lower than in cells transfected with non-specific siRNA (−si; Fig. 3, panel 3, lane 3).

Treatment of the triple-negative breast cancer cells with 10^−6^ M 4-hydroxytamoxifen stimulated the phosphorylation of the EGF-receptor more strongly than it was observed after treatment with 17β-estradiol (Fig. 3, panel 3, lane 4). Phosphorylation of EGF-receptor by 4-hydroxytamoxifen was almost completely abolished in cells transfected with siRNA directed against GPR30 (+si; Fig. 3, panel 3, lane 5).

The analysis of GPR30 signaling in the cell line HCC1806 showed similar consequences of the knock-down of GPR30 mRNA (data not shown).

### Expression of *c*-*fos* protooncogene in response to 17β-estradiol and 4-hydroxytamoxifen

Expression of *c*-*fos* is a prerequisite of proliferation. Therefore, *c*-*fos* expression was analyzed in two triple-negative cell lines after treatment with either 17β-estradiol or 4-hydroxytamoxifen. Upregulation of *c*-*fos* expression by these two compounds in cells transfected with control siRNA (−si) was compared with *c*-*fos* expression of cells pretreated for 7 days with siRNA against GPR30 (+si).

**Fig. 4:**
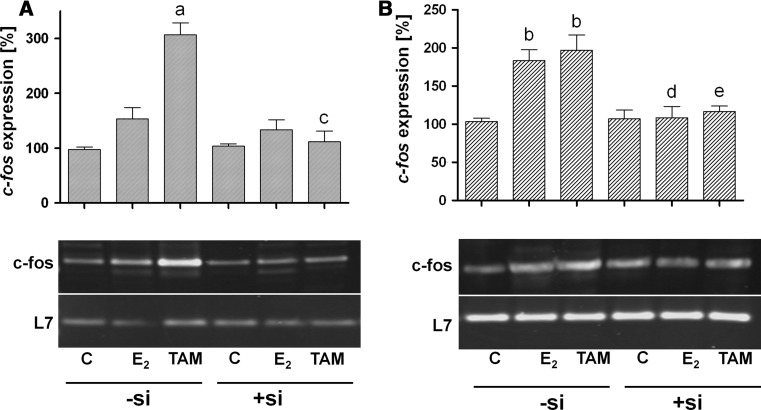
Prevention of *c*-*fos* expression by the knock-down of GPR30. Triple-negative breast cancer cell lines, **a** MDA-MB-435 and **b** HCC1806, transfected with non-specific siRNA (control) or with GPR30siRNA were stimulated for 30 min either with 10^−9^ M 17β-estradiol or 10^−6^ M 4-hydroxytamoxifen. mRNA was extracted, transcribed into cDNA, and amplified by PCR using primers specific for *c*-*fos*. L7, a ribosomal housekeeping gene, was amplified to prove the presence of equal amounts of RNA in each PCR reaction of the respective cell line. Representative results of three separate experiments. (*a*) *p* < 0.001 versus control, (*b*) *p* < 0.01 versus control, (*c*) *p* < 0.001 versus TAM, −si, (*d*) *p* < 0.01 versus E2, −si, (*e*) *p* < 0.01 versus TAM, −si

In control cells of the cell line MDA-MB-435 (−si), expression of *c*-*fos* clearly increased after 30 min stimulation with 10^−9^ M 17β-estradiol (1.5-fold) as well as 10^−6^ M 4-hydroxytamoxifen (3.1-fold, *p* < 0.001) (Fig. 4a). Inactivation of GPR30 led to an almost complete inhibition of *c*-*fos* induction (Fig. 4a, lanes 5 and 6).

In HCC1806 cells, 17β-estradiol stimulated *c*-*fos* expression about 1.8-fold (*p* < 0.01) and 4-hydroxytamoxifen led to an about twofold increase (*p* < 0.01) of *c*-*fos* expression (Fig. 4b). Suppression of GPR30 expression using siRNA blocked the induction of *c*-*fos* by both compounds in HCC1806 cells similar to the effects observed in MDA-MB-435 cells (Fig. 4b, lanes 5 and 6).

## Discussion

The clinical outcome for patients with triple-negative breast cancer is still poor, despite intensive chemotherapy using cytotoxic drugs like platinum compounds. A search for more specific, targeted therapeutic options has been performed in recent years. In particular, PARP inhibitors were applied, taking advantage of a disturbed DNA-repair due to frequent BRCA1-mutations present in triple-negative breast cancer [4, 5]. Other investigators have chosen the frequently observed overexpression of the EGF-receptor as target using EGF-R antibody Cetuximab for a tailored therapy [3].

In the present study, we investigated the possible role of the GPR30, in the growth stimulation of triple-negative breast cancer cells. Immunohistochemical staining of sections from triple-negative breast tumors revealed that almost all tumors were strongly positive for GPR30 (unpublished results). GPR30 was also found to be strongly expressed in the two triple-negative cell lines under investigation. Therefore, GPR30 expression is a feature frequently occurring in triple-negative breast cancer that might be used for targeted therapy.

Filardo et al. [11] intensively examined the role of GPR30 in response to 17β-estradiol and unraveled the signaling pathway downstream of this receptor. Ligands binding to GPR30 induce the dissociation of the heterotrimeric G-proteins. The βγ-subunit activates the tyrosine kinase Src. Subsequently, EGF-receptor is autophosphorylated at tyrosine^1173^ initiating the ras-MAP-kinase signaling, finally inducing proliferation of estrogen-stimulated cells independent of ERα [8, 9]. Most experiments elucidating the signaling pathways of GPR30 were performed with the breast cancer cell line SK-Br3 lacking expression of ERα and ERβ. However, this SK-Br3 cell line does not represent a triple-negative breast cancer cell line, as SK-Br3 cells overexpress Her2, whereas triple-negative breast cancer cells lack overexpression of human EGF-receptor 2 (Her-2) [18].

In contrast, many triple-negative breast tumors overexpress the EGF-receptor (Her-1) [19]. EGF is able to induce the expression of GPR30 [20]. 17β-Estradiol activates the cytosolic kinase Src via GPR30 and Src activates matrix metalloproteases that release EGF from extracellular matrix. The subsequent induction of GPR30 expression leads to a positive feedback loop that boosts the induction of proliferation by 17β-estradiol in triple-negative breast tumors.

In this report, we present evidence that in the triple-negative breast cancer cells studied, all necessary steps of the GPR30 signaling are activated in response to 17β-estradiol and 4-hydroxytamoxifen. Src phosphorylation is increased in both cell lines and EGF-receptor is phosphorylated at Tyr^1173^ after stimulation with both estrogenic compounds. Both kinases are more strongly activated by 4-hydroxytamoxifen than by 17β-estradiol. The activation of these kinases was completely prevented in the triple-negative cell lines by knock-down of GPR30.

Some controversy exists about the nature of the estrogen receptor that is responsible for the rapid non-genomic effects of 17β-estradiol. Whereas some authors concluded that the activation of Src kinase, EGF-receptor, and the MAP-kinase Erk1/2 in breast cancer cells lacking ERα is elicited by GPR30 [11, 12], other authors predominantly hypothesize the presence of a truncated ERα that localizes at the membrane supported by the scaffolding protein caveolin [10].

Opposite to this, the results of our experiments provide further evidence that the non-genomic effects of 17β-estradiol, like activation of c-Src, phosphorylation of the EGF-receptor, and increased expression of *c*-*fos*, are dependent on the presence of GPR30. As we describe in this report, all these non-genomic effects of 17β-estradiol are detectable in the two triple-negative breast cancer cell lines and inactivation of GPR30 by means of siRNA prevent the activation of all pathways described above. Therefore, this report proves without doubt that GPR30 mediates the non-genomic effects of 17β-estradiol.

From the clinical point of view, the results presented in this report highlight GPR30 as an important new target for a more specific therapy of triple-negative breast cancer.

There have already been described some compounds that specifically inhibit GPR30. Estriol binds to GPR30 and inhibits GPR30 signaling weakly as shown for the upregulation of *c*-*fos* expression [21]. A substituted dihydroquinoline, G15, was identified that binds to GPR30 with an affinity of 20 nM. G15 was able to effectively block calcium mobilization by 17β-estradiol in the GPR30 expressing breast cancer cells SKBr3 [22]. Despite this, there is still a need to search for more effective inhibitors of GPR30 possessing a higher affinity and good bioavailability, before a therapy of triple-negative breast cancer targeting GPR30 may enter clinical trials.

## Abbreviations

EGF: Epidermal growth factor
ERα: Estrogen receptor α
Erk: Extracellular signal-regulated kinase
FCS: Fetal calf serum
GPR30: G-protein coupled receptor
RT-PCR: Reverse transcription polymerase chain reaction
PARP: Poly-(ADP-ribose) polymerase
